# Implications of the Gut Microbiota for Brain Function and Behavior in Schizophrenia

**DOI:** 10.7759/cureus.64340

**Published:** 2024-07-11

**Authors:** Ubaid Ansari, Fatima Ansari, Dawnica Nadora, Arman Omid, Alexi Omid, Meraj Alam, Denise Nadora, Forshing Lui

**Affiliations:** 1 Neurology, California Northstate University College of Medicine, Elk Grove, USA; 2 Psychology, Mission College, Santa Clara, USA; 3 Dermatology, California Northstate University College of Medicine, Elk Grove, USA; 4 Gastroenterology, California Northstate University College of Medicine, Elk Grove, USA; 5 Psychiatry, California Northstate University College of Medicine, Elk Grove, USA; 6 Clinical Sciences, California Northstate University College of Medicine, Elk Grove, USA

**Keywords:** prebiotics, probiotics, dysbiosis, gut-brain axis, gut microbiota, schizophrenia

## Abstract

Schizophrenia is a severe, chronic psychiatric disorder characterized by delusions, hallucinations, cognitive impairments, and emotional dysregulation. This psychiatric illness is often resistant to treatment. This literature review aims to analyze the relationship between this complex psychological disorder and the gut microbiota found within the human body. The brain and gut are interconnected, and emerging research suggests a link between gut dysbiosis and schizophrenia. Gut dysbiosis refers to an imbalance or disruption in the composition and function of the gut microbiome. The studies comparing the gut microbiota of patients with schizophrenia to those without highlight significant differences at the phylum and genus levels, providing evidence of gut microbiome alteration. The lack of diversity of microbiota in schizophrenia patients can be altered and improved to a healthier microbiome by way of dietary intervention. Interventions that target the gut-brain axis, such as dietary probiotics or prebiotics, may help alleviate certain symptoms of schizophrenia and help improve patients’ well-being. Understanding the complex interplay between gut microbiome health and schizophrenia may allow for the development of targeted interventions that alter the gut microbiome of patients with schizophrenia and, in turn, mitigate their symptoms and improve their quality of life.

## Introduction and background

Schizophrenia is a complex chronic illness characterized by a range of cognitive, emotional, and behavioral symptoms that heavily impact an individual’s quality of life. This multifactorial disorder causes a decline in functioning and psychosis [1]. Patients with schizophrenia have both positive and negative symptoms. Positive symptoms encompass hallucinations, delusions, disorganized thought or speech, as well as heightened perceptions or actions [1]. Negative symptoms involve social withdrawal or anhedonia and may indicate a deficiency in typical brain functioning [1]. Traditional approaches to understanding and treating schizophrenia have primarily focused on neurochemical and genetic factors. Scientists have historically studied the brain to better understand the pathophysiology of schizophrenia. However, recent research suggests that the gut, which contains trillions of microbes, might also play a significant role in disease pathogenesis.

Psychological disorders such as depression, anxiety, and autism spectrum disorders have been found to correlate with gastrointestinal disruptions, and gastrointestinal diseases often present with psychological disorders, which are commonly linked to changes in the gut microbiome [2]. This literature review underscores the growing importance of understanding the gut-brain axis in schizophrenia, as schizophrenia patients may have altered gut microbiomes, and aims to synthesize emerging research in the field. The gut-brain axis involves connecting the central and enteric nervous systems, linking both the emotional and cognitive brain centers with intestinal functions throughout the body axis [3]. This bidirectional communication network has garnered significant attention for its potential involvement in mental health. This axis encompasses neural, hormonal, and immunological pathways, facilitating interactions between gut microbiota and brain function [4]. In recent years, numerous studies have demonstrated that alterations in gut microbiota composition can influence brain chemistry, inflammation, and behavior, raising intriguing possibilities for novel therapeutic approaches in psychiatric conditions, including schizophrenia [4]. There is converging evidence from human and animal studies leading to the conclusion that gut dysbiosis potentially plays a role in developing schizophrenia. This review aims to determine if there is a link between dysbiosis and schizophrenia or if the disease results in dysbiosis, as well as review how those alterations in the gut might be treated by way of probiotics and prebiotics.

Understanding the role of gut microbiota in schizophrenia not only broadens the scope of psychiatric research but also opens new avenues for innovative treatment strategies. This literature review aims to synthesize current findings on the implications of gut microbiota for brain function and behavior in individuals with schizophrenia. By examining the intricate relationships between gut microorganisms and various aspects of brain health, this review seeks to elucidate potential mechanisms through which gut dysbiosis may contribute to the onset and progression of schizophrenia. Additionally, this review will explore the therapeutic potential of microbiota-targeted interventions, such as probiotics, prebiotics, and dietary modifications, in mitigating schizophrenia symptoms and improving patient outcomes.

## Review

Gut dysbiosis in schizophrenia

Dysbiosis occurs when there is a disruption to the microbiome, resulting in an imbalance in the microbiota of the gut [5]. One major emerging aspect of gut dysbiosis in schizophrenia is gut microbiota playing an important role in a person’s early development and neural maturation [5]. For example, prenatal microbial infections can increase the risk of developing schizophrenia by 10-20 times [6]. Dysbiosis is characterized by an imbalance in gut microbial composition and can result from factors such as diet, lifestyle, stress, and medications, causing disruptions in overall host health [5]. Dysbiosis-induced intestinal permeability, bacterial translocation, and inflammation are linked to neurological impairments and autoimmune responses seen in schizophrenia, potentially contributing to the gastrointestinal dysfunctions often observed in affected individuals [5]. One can think of the human gut as a community, and dysbiosis would subsequently be any disturbances to the microbial residents; this alteration accounts for an inadequate education of the host’s immune system and leads to immune-mediated disease [7]. Schizophrenia is indeed linked to abnormalities in the immune system and subsequent neuroinflammation.

Alterations in the gut microbiome

Alterations in the gut microbiome differentiate those with schizophrenia and those without. An empirical study by Nguyen et al. in 2019 showed that there is an altered gut microbiome in individuals who have schizophrenia as opposed to those who do not. The researchers gathered stool samples and assayed them utilizing 16S rRNA sequencing of the V4 region. Analysis of the stool samples from 25 schizophrenia subjects and 25 control subjects indicated significant differences in the microbiome composition of the two cohorts [8]. Specifically, *Proteobacteria* were found to be comparatively lower in individuals with schizophrenia at the phylum level. Moreover, it was determined that at the genus level, *Anaerococcus* showed a relative increase in schizophrenia cases, while *Haemophilus*, *Sutterella*, and *Clostridium* bacteria were reduced. In individuals with schizophrenia, higher levels of *Ruminococcaceae* were associated with milder negative symptoms, whereas elevated *Bacteroides* levels correlated with more severe depressive symptoms. Additionally, a higher abundance of *Coprococcus* was linked to an increased risk of developing coronary heart disease [8]. This study provided evidence of gut microbiome alteration in individuals with schizophrenia versus those without. However, there are some pathways to help enhance this study’s significance. One way is to have larger subject groups. In addition, a longitudinal study would allow researchers to investigate the subjects’ gut microbiome over time and allow for more specific insights.

In a study comparing participants with schizophrenia to healthy individuals, significant differences in gut bacteria were found [9]. Specific types of bacteria, such as bacterial families Aerococcaceae and Rikenellaceae, were strongly linked to the severity of schizophrenia [9]. Using these bacteria, researchers were able to distinguish between people with schizophrenia and healthy individuals accurately. The gut bacteria in subjects with schizophrenia were less diverse compared to healthy individuals, which could indicate an unhealthy gut [9]. According to the researchers, this lack of diversity is unique to schizophrenia. Certain families of gut bacteria that are important for gut health were found to be reduced in people with schizophrenia [9]. In the same study, another experiment was conducted with mice by transferring gut bacteria from people with schizophrenia to mice, which led to changes in their gut bacteria and schizophrenic behavior. These behavioral changes in mice are consistent with other known models of schizophrenia that involve glutamate disruption [9]. The study highlighted that the mice also showed disruptions in brain chemistry related to glutamate, which is a major factor in the development of schizophrenia. The researchers stated that the specific brain regions affected aligned with those implicated in schizophrenia, suggesting a targeted effect from the transferred gut bacteria.

Additionally, the study underscored that changes in lipid metabolism were observed in the mice that received gut bacteria from people with schizophrenia. This aligns with prior research conducted among schizophrenia patients, where lipid disturbances are common [9]. These findings suggest that gut bacteria play a role in schizophrenia-related behaviors and brain chemistry. However, it is important to note that most patients in the study were taking antipsychotic medication, which could have influenced the results [9].

Gut microbiota differences in schizophrenia and metabolic syndrome

In a study conducted by Thirion et al. (2023), a research team conducting a large study on the relationship between schizophrenia and altered gut microbiomes found that schizophrenia gut microbiota was different from their healthy control group [9]. There were 132 schizophrenia patients with increased waist circumference compared to two control groups: one consisting of 132 healthy individuals matched for age and sex, and another consisting of 132 individuals with metabolic syndrome matched for age and sex [9]. A unique aspect of this study was its control group of individuals with metabolic syndrome. This syndrome consists of a cluster of the following metabolic abnormalities: hypertension, insulin resistance, obesity, fatty liver, and cardiovascular diseases [9]. One of the most beneficial components of this study is that it controlled for the possible confounder of metabolic syndrome. This syndrome can also affect the gut, so researchers could differentiate between the gut alterations in schizophrenia and metabolic syndrome by controlling for this variable. The study indicated that individuals with schizophrenia have reduced gut species richness compared with both control groups [9].

Role of nutrition in treating schizophrenia patients

Schizophrenia is one of the most disabling chronic conditions, often with treatment-resistant symptoms [10]. Negative symptoms such as emotional blunting and apathy, along with cognitive impairment, are particularly resistant to antipsychotic drugs [10]. Given the limited benefits and potential drawbacks of current treatments due to side effects and tolerance, there is a growing interest in exploring the impact of nutrition on mental disorders, as the connection between nutrition and mental health is linked to neurotransmitter and hormonal pathways in the gut that influence brain function [10]. Research is being conducted into anti-inflammatory diets or Mediterranean diets for schizophrenia, as there is an association between inflammation and schizophrenia. Diet is said to have a lasting impact on gut microbiome alteration and cognition. One study focussed primarily on probiotics and prebiotics as they can help restore the lost diversity of gut bacteria [5]. Gut dysbiosis is one of many factors that can cause schizophrenia, and one hypothesis is that eating food rich in bacteria that is seemingly lacking in schizophrenia patients can potentially increase the diversity of the gut microbiome. Using pro and prebiotics regularly is often recommended to keep gut bacteria balanced and strengthen the body’s immune system naturally [5]. However, more thorough studies are needed to determine how well they work long-term, which specific bacteria are the most helpful, and how much fiber or prebiotics are needed to grow. Prebiotics and probiotics strengthen the effect of one another [5]. Targeted alteration of schizophrenia patient’s gut bacteria may serve as a promising avenue for treatment, alongside any prescribed medication [5].

Impact of probiotic supplementation on schizophrenia patients

Probiotics are live microorganisms that confer health benefits when consumed in adequate amounts [11]. Probiotics have been shown to decrease depressive and anxiety symptoms in schizophrenia patients [12]. In one single open-arm study on probiotics, 29 outpatient schizophrenia participants were treated with *Bifidobacterium breve* strain A-1 at a dose of 1,011 cfu/day [12]. The four-week treatment course and equal observation period (four weeks) aimed to explore the probiotic’s impact on anxiety and depressive symptoms in schizophrenia patients. Specific improvements were noted in all patients’ Hospital Anxiety and Depression Scale (HADS) and Positive and Negative Syndrome Scale (PANSS) scores, with 12 responders showing notable reductions in HADS scores, which included more than a 25% decrease from baseline [12]. Moreover, there were changes in gut microbiome composition and increased expression of certain immune markers such as interleukin (IL)-22 and tumor necrosis factor-related activation-induced cytokine in responders [12]. The authors noted that one possible limitation was that they could not exclude a placebo effect. This research reveals that there is possibly a link between probiotics improving depressive symptoms and anxiety in schizophrenia patients.

Vitamin D used in tandem with probiotics has beneficial impacts on schizophrenia patients [10]. Vitamin D is a well-known vitamin that plays a role in healthy bone and calcium homeostasis [13]. Vitamin D also has been shown to have anti-inflammatory properties. It has been shown to decrease the serum C-reactive protein level and stabilize IL-10 level [14]. Vitamin D deficiency is prevalent among schizophrenia patients and is associated with environmental risk factors and comorbid conditions such as obesity, insulin resistance, and cardiovascular diseases [13]. In one study, 60 patients with chronic schizophrenia were randomly assigned to receive either 50,000 IU of vitamin D3 every two weeks along with a daily dose of 8 × 10^9^ CFU of probiotics (n = 30) or a placebo (n = 30) over 12 weeks [10]. The administration of probiotics and vitamin D for 12 weeks to individuals with chronic schizophrenia positively impacted the overall and total PANSS scores and metabolic profiles [10].

Interestingly, probiotics are present in a variety of foods worldwide [15]. It would be beneficial for future studies to focus on the roles that different supplements and cultural foods containing probiotics have on schizophrenia symptoms. For example, kombucha is a fermented tea that contains probiotics. Similarly, there are dishes such as kimchi or sauerkraut that are made of fermented cabbage or ingredients such as miso, sourdough bread, or apple cider vinegar that contain probiotics [15].

Impact of prebiotic supplementation on schizophrenia patients

Prebiotics are dietary fibers that promote the growth and activity of beneficial bacteria in the gut, which, in turn, can positively affect various aspects of health [11]. These fibrous substrates are present in vegetables, nuts, and fruits, to name a few [5]. The consumption of prebiotics by schizophrenia patients may allow for cognitive benefits and play an important role as an indicator in the recovery of schizophrenia patients [16]. A study in rats similarly indicated that prebiotics entail greater flexibility in cognition. The rats that were fed prebiotics were able to shift between an intra-dimensional to an extra-dimensional set in a smaller number of trials than the control group [17]. Prebiotic supplementation may potentially benefit schizophrenia by promoting the growth of beneficial gut bacteria, which can modulate the gut-brain axis [18]. This modulation may help reduce inflammation, improve neurotransmitter balance, and enhance overall brain function, potentially alleviating some symptoms of schizophrenia [18]. Research into prebiotics as a complementary treatment aims to harness these effects to support traditional therapies and improve patient outcomes in schizophrenia. It is important to note that there are not as many studies on prebiotics as there are on probiotics; however, any restored microbial diversity is sustained by schizophrenia patients consuming prebiotics because they propagate and allow for gut microbiota activity. Furthermore, a lack of dietary fiber leads to dysbiosis, which can be linked to diseases and psychological disorders [5].

Discussion

This literature review aimed to examine the relationship between gut microbiota and schizophrenia, delving into how gut dysbiosis could be a factor in causing schizophrenia or worsening the symptoms. The research found that there is an association between mental health disorders and dysbiosis. This literature review strongly suggests that the gut microbiome plays a role in psychological disorders, providing valuable insights into new avenues for managing schizophrenia through nutritional interventions such as probiotics and prebiotics. The analysis of gut microbiota in schizophrenia patients versus healthy controls revealed differences in the gut microbiome composition. Variations were in bacterial families such as *Proteobacteria*, *Haemophilus*, *Sutterella*, *Clostridium*, *Ruminococcaceae*, *Bacteroides*, and *Coprococcus* [8]. A large-scale study highlighted that most human gut microbiota come from the following phyla: *Bacteroidetes*, *Actinobacteria*, *Firmicutes*, and *Proteobacteria* [19]. *Proteobacteria* is a major gastrointestinal bacteria, yet the amount is decreased in individuals with schizophrenia [8].

The human studies concluded that there is a difference in the gut microbiome between the control groups and schizophrenia patients. However, the placebo effect and other possible lifestyle confounding factors cannot be ruled out completely. Many studies showed significant differences between the two cohorts. One study notably controlled for the confounding factor of metabolic syndrome, which could be comorbid with schizophrenia. The researchers were able to distinguish gut alterations successfully. Furthermore, the study found that gut microbiota’s functional potential explained cognitive variability of 11%. Thus, their results claimed that targeting gut microbiota through interventions could alleviate the cognitive dysfunction seen in schizophrenia. This aligns with the results of the 2019 study done by Kao et al. where they found that targeting gut microbiota in schizophrenia patients by consumption of prebiotics results in cognitive benefits.

Human studies were only able to establish there is an association between gut dysbiosis and schizophrenia, creating one clear line of evidence, another one of which comes from animal studies. The study with mice showed that the transfer of schizophrenia gut microbiota to healthy mice results in behavioral changes that align with the presentation of schizophrenia, glutamate disruptions, and changes in lipid metabolism [20]. This finding underscores that alterations in the gut microbiome lead to changes in brain function and behavior. Animal studies and human studies created converging evidence for gut dysbiosis being linked to schizophrenia.

However, there are limitations in the studies. For one, there is a need for larger-scale human studies. Small sample sizes reduce statistical significance and make it difficult to draw meaningful conclusions. Furthermore, studies should attempt to control for confounding factors associated with metabolic changes such as metabolic syndrome, cardiovascular disease, smoking, and other possible factors. One study in this literature review controlled for metabolic factors, which made it more significant. Medication also served as a confounding factor. Recent research even suggests that schizophrenia medication may play a role in gut alteration, although more research is needed in this area. Clozapine is a drug used to treat treatment-resistant schizophrenia [21]. One study reported that the experimental group demonstrated improved overall functioning following clozapine therapy [21]. While the therapy was generally well-tolerated, some moderate side effects were noted; suicidal thoughts decreased with clozapine treatment, and there was a notable reduction in both negative symptoms and general psychopathology [21]. With previous research, clozapine seemed to be a promising treatment. However, a recent study by Vasileva et al. consisting of 97 adults revealed that the link between the gut and schizophrenia is related to treatment resistance and clozapine use. According to the study, the link is not connected to age, lifestyle, or side effects from medication such as constipation or metabolic syndrome. This indicates that treatment-resistant schizophrenia patients have a significantly different gut microbiome than that of treatment-responsive patients and individuals without schizophrenia [21]. Although the study could not rule out existing differences in gut microbiome composition, it concluded that clozapine can induce gut alterations. While this literature review examined gut dysbiosis as a cause of schizophrenia, gut dysbiosis as a result of schizophrenia medication is a nuanced and promising area of research for future studies.

Although the evidence of probiotics and prebiotics for schizophrenia patients sheds light on some potential treatment pathways, more studies are required. Future research should focus on how increasing or lowering the dosage of prebiotics and probiotics impacts the gut microbiome and patients’ negative or positive symptoms. Although there are promising studies in the field of probiotics and prebiotics, they may serve as a supplement to schizophrenia treatment and not as a sole treatment option. There is much to look forward to in this promising field as emerging research investigated in this review has already provided valuable insights.

## Conclusions

Schizophrenia is a life-altering, chronic condition, often with symptoms that are resistant to treatment. By exploring the role of gut microbiota and the pathophysiology of schizophrenia, this literature review aims to mitigate outcomes for schizophrenia patients and potentially incentivize future research to contribute to this constantly evolving field. Microbial infections seem to play a significant role in schizophrenia pathogenesis at multiple stages of life. During fetal development, microbial infection may increase the likelihood of developing schizophrenia. Various studies have also found specific differences in gut microbiome and gut bacteria in healthy individuals compared to those with schizophrenia. One study noted that gut microbiome diversity and richness are lower in individuals with schizophrenia compared to healthy individuals and those with metabolic syndrome. Furthermore, this research highlights the critical role of diet in influencing gut microbiome in individuals with schizophrenia and details the ability of probiotics and prebiotics to help alleviate symptoms due to their synergistic effect in reducing inflammation, as well as depressive and anxiety symptoms. Future research is needed to enrich the overall understanding of the impact of structured nutritional or microbial therapies on the gut-brain axis. While this literature review highlighted the beneficial impact of the combination of vitamin D and probiotics on schizophrenia patients, there may be other potential combinations of nutrients that provide valuable outcomes. Moreover, larger longitudinal studies on targeted microbiome interventions are essential in recognizing dynamic interactions between gut microbiota and schizophrenia. The knowledge gained from this research on the potential factors affecting the gut microbiota and schizophrenia opens new avenues for targeted interventions in the gut-brain axis, which may serve as potential treatments for various psychological and neurological disorders beyond schizophrenia.
